# siRNA-Loaded Hydroxyapatite Nanoparticles for *KRAS* Gene Silencing in Anti-Pancreatic Cancer Therapy

**DOI:** 10.3390/pharmaceutics13091428

**Published:** 2021-09-08

**Authors:** Dandan Luo, Xiaochun Xu, M. Zubair Iqbal, Qingwei Zhao, Ruibo Zhao, Jabeen Farheen, Quan Zhang, Peiliang Zhang, Xiangdong Kong

**Affiliations:** 1 Institute of Smart Biomedical Materials, School of Materials Science and Engineering, Zhejiang Sci-Tech University, Hangzhou 310018, China; 201810301008@mails.zstu.edu.cn (D.L.); 201820201066@mails.zstu.edu.cn (X.X.); zubair@zstu.edu.cn (M.Z.I.); rzhao@zstu.edu.cn (R.Z.); jabeenfarheen87@zstu.edu.cn (J.F.); quanzhang@zstu.edu.cn (Q.Z.); 2 Zhejiang-Mauritius Joint Research Center for Biomaterials and Tissue Engineering, Zhejiang Sci-Tech University, Hangzhou 310018, China; 3 School of Textile Science and Engineering, Zhejiang Sci-Tech University, Hangzhou 310018, China; 4 Research Center for Clinical Pharmacy & Key Laboratory for Drug Evaluation and Clinical Research of Zhejiang Province, The First Affiliated Hospital, Zhejiang University School of Medicine, Hangzhou 310003, China; qwzhao@zju.edu.cn; 5 Department of Radiation Oncology, Linyi Central Hospital, Linyi 276400, China; zpl6@sohu.com

**Keywords:** hydroxyapatite, siRNA delivery, pancreatic cancer cells, *KRAS* gene, gene silence, anticancer

## Abstract

Pancreatic carcinoma (PC) is greatly induced by the *KRAS* gene mutation, but effective targeted delivery for gene therapy has not existed. Small interfering ribonucleic acid (siRNA) serves as an advanced therapeutic modality and holds great promise for cancer treatment. However, the development of a non-toxic and high-efficiency carrier system to accurately deliver siRNA into cells for siRNA-targeted gene silencing is still a prodigious challenge. Herein, polyethylenimine (PEI)-modified hydroxyapatite (HAp) nanoparticles (HAp-PEI) were fabricated. The siRNA of the *KRAS* gene (siKras) was loaded onto the surface of HAp-PEI via electrostatic interaction between siRNA and PEI to design the functionalized HAp-PEI nanoparticle (HAp-PEI/siKras). The HAp-PEI/siKras was internalized into the human PC cells PANC-1 to achieve the maximum transfection efficiency for active tumor targeting. HAp-PEI/siKras effectively knocked down the expression of the *KRAS* gene and downregulated the expression of the Kras protein in vitro. Furthermore, the treatment with HAp-PEI/siKras resulted in greater anti-PC cells’ (PANC-1, BXPC-3, and CFPAC-1) efficacy in vitro. Additionally, the HAp-PEI exhibited no obvious in vitro cytotoxicity in normal pancreatic HPDE6-C7 cells. These findings provided a promising alternative for the therapeutic route of siRNA-targeted gene engineering for anti-pancreatic cancer therapy.

## 1. Introduction

Pancreatic carcinoma (PC) is the most severe type of human melanoma that caused secondary neural microenvironment and has the highest mortality among all diagnosed tumors [1,2,3]. The available treatments such as chemotherapy combined with radiotherapy can only enhance 5.0–70 months, and a 5-year survival rate is about 4% in PC patients [4,5,6]. Even for some patients diagnosed at early stages and who received intensive treatment the 5-year survival rate is lower than 20% [7,8]. Additionally, the mortality rate (48,220) of PC patients is nearly close to the rate of incidence (60,430) due to the absence of effective treatment. Therefore, advanced therapy is essential in uncovering the disease in its infancy [9]. Further, several factors are involved in PC induction and progression, and among them *KRAS* proto-oncogene activation mutation is the most crucial in diagnosed patients [10]. Almost 95% of cases of PC have point mutation at G12, G13, and Q61 codons in the *KRAS* activation domain as a result of the mutant Kras protein generation, which provokes growth and proliferation of pancreatic tumors [11,12]. However, *KRAS*-based targeting is a challenging assignment. Thus, improvement in PC diagnosis, treatment, and prevention requires interdisciplinary cooperation and novel trial designs [13].

Recently, RNA interference is considered a promising therapeutic alternative in the treatment of PC. It is the most sophisticated post-transcriptional gene silencing therapy by small interfering double-stranded RNA (siRNA) that can induce sequence-specific silencing of about 80% of the targeted protein’s mRNA to effectively inhibit tumor cell proliferation [14,15,16,17]. Further, it is an emerging genetic technique that deals with several kinds of human melanomas. For instance, in *KRAS*-dependent mutant cancer, dual knockout of RhoA/Rho kinase (ROCK) and polp-like kinase-1 by siRNA enhanced the expression of the cyclin-dependent kinase, which ultimately impaired the growth of *KRAS*-mutant cells’ proliferation [18]. Guan et al. [19] successfully induced apoptosis of PC2 PC cell lines by inhibiting the expression of the apoptosis-related gene *Survivin* using RNA interference technology. However, siRNA is a hydrophilic biological macromolecule with a molecular weight of about 1.3 × 10^4^, along with a large size and high negative surface charge, which makes it difficult to cross the same electronegative cell membrane [20]. Meanwhile, free siRNA cannot escape from the extensive cell nuclei in the bloodstream and active renal clearance, which makes it almost impossible for exogenous free siRNA existence to conduct RNA interference independently [21,22]. Therefore, the development of a low-toxicity and high-efficiency carrier system that accurately delivers siRNA into the cells is still a huge challenge for siRNA-based targeted gene silencing.

Nano-hydroxyapatite (nHAp), a naturally occurring calcium and phosphorus-based inorganic material, is currently widely used for biomedical applications [23]. nHAp showed appealing possibilities in tissue engineering due to its unique physio-chemical features. A study was reported regarding the suitability of Fe/Sr co-substituted HAp nanoparticles for tissue engineering. These nanoparticles were appropriated for human mesenchymal cells and demonstrated high blood compatibility, enhanced drug release capability, and an improved tissue-repair profile [24]. The previous study reported that a HAp-laminin composite showed the optimal neural stem cells (NSCs) adhesion and well-spread cell morphology, and also supported NSCs proliferation without causing any cytotoxicity, which is promising for neural-related applications [25]. Additionally, nHAp has aroused extensive interest in drug delivery research due to its high biocompatibility, low cytotoxicity, better biodegradability, and favorable affinity to plenty of molecules such as chemotherapy drugs, pDNA, and siRNA. In particular, it gained remarkable potential as a gene delivery system in tumor treatment and therapy [26]. Gu et al. [27] reported that the NR2B siRNA-loaded HAp nanoparticle could not only eliminate the formalin-induced tetanic response but also significantly downregulate the expression of NR2B protein in mice. However, nHAp has weak positive surface charges that lead to a feeble loading and low transfection ability of siRNA [28]. Therefore, modification in surface charges is crucial for siRNA-targeted gene silencing, which can be achieved by developing a simple and effective modification strategy.

Earlier reports were concordant with *KRAS* activation mutation and highly significantly associated with the development of metastasis pancreatic carcinoma [29]. Recently, a siRNA-mediated knockout mechanism was implicated in the regulation of protein overexpression [30]. Therefore, *KRAS* silencing-based siRNA might provide a promising option for the treatment of pancreatic cancer.

In this study, HAp nanoparticles were prepared by a chemical precipitation method and modified by polyethyleneimine (PEI), a commonly used cationic polymer. PEI-modified HAp nanoparticles (HAp-PEI) were used as the carrier of *KRAS* siRNA. The loading and transfection ability of siRNA was examined by agarose gel electrophoresis and observed by confocal microscope, respectively. The biocompatibility, as well as the effect of pancreatic cancer-related gene silencing and cancer-cell inhibition after carrying siRNA were also investigated.

## 2. Materials and Methods

### 2.1. Materials

Ca(NO_3_)_2_, Na_2_HPO_4_, NaOH, polyethylene glycol-400 (PEG-400) and polyethyleneimine (PEI, M.W. 70,000) were purchased from Aladdin (Shanghai, China). We purchased 3-(4,5-Dimethyl-2-thiazolyl)-2,5-diphenyl-2H-tetrazolium bromide (MTT), Trypsin-EDTA solution, Lysotracker^TM^ Red and the QuantiChrom^TM^ calcium ion detection kit from Beyotime Institute of Biotechnology (Shanghai, China). High glucose Dulbecco’s modified eagle medium (DMEM) and Roswell park memorial institute-1640 (RPMI-1640) were obtained from BioInd (Beit-Haemek, Israel). Fetal bovine serum (FBS) was purchased from Gibco (Sidney, Australia). Fluorescently labeled siRNA-FAM, negative control siRNA (siNC, sense strand, 5′-UUC UCC GAA CGU GUC ACG UTT-3′), *KRAS* siRNA (siKras, sense strand, 5′-CAC CAU UAU AGA GAA CAA ATT-3′), and commercial transfection reagent siRNA-Mate were obtained from Genpharm (Shanghai, China). Other chemicals and reagents were of analytical grade.

Human pancreatic cancer cells PANC-1, BXPC-3, CFPAC-1, and human pancreatic ductal epithelial cell HPDE6-C7 were purchased from the cell bank of Chinese Academy of Science (Shanghai, China). PANC-1 and CFPAC-1 were grown in high glucose DMEM, and HPDE6-C7 were maintained in RPMI-1640 medium. Cells were incubated with 10% FBS and 1% penicillin/streptomycin at 37 °C in a 5% CO_2_ atmosphere.

### 2.2. Synthesis and Characterization of HAp and HAp-PEI

HAp nanoparticles were synthesized as following: 10 mL 3.74 mmol/L (NH_4_)_2_HPO_4_ aqueous solutions were added dropwise to 10 mL 6.25 mmol/L Ca(NO_3_)_2_, and the pH was adjusted to 10~13 by adding ammonia solution. After incubation for 30 min, 100 μL dispersant PEG-400 was added to the suspension and incubated for another 3 h. The nanoparticle suspension was centrifuged (8000 rpm × 3 min), and HAp precipitate was collected.

Then, 100 μL of 10 mg/mL PEI was added to the HAp suspension to synthesize HAp-PEI nanoparticles. After 3 h, the mixture was centrifuged at 8000 rpm for 3 min to remove the residual PEI and obtained HAp-PEI precipitate.

The morphology, structure, and chemical composition of HAp and HAp-PEI were characterized by field emission scanning electron microscope (FE-SEM), transmission electron microscope (TEM), dynamic light scattering (DLS), X-ray diffraction measurement (XRD), fourier transform infrared spectroscopy (FTIR), Raman spectroscopy, thermogravimetry (TG), and Energy Dispersive Spectrometer (EDS).

### 2.3. In Vitro Degradation of HAp-PEI

The 90 mg of HAp-PEI was incubated in 10 mL of PBS (pH value of 5.6, 6.5 and 7.4) under gentle shaking at 37 °C for various times up to 90 days. The pH value of 5.6, 6.5 and 7.4 was used to simulate human cell lysosome, tumor and normal fluid microenvironment, respectively. At specific time points, the supernatant was withdrawn by centrifugation and replaced by the same volume of fresh PBS. The concentration of Ca^2+^ in the clear supernatant was detected by the QuantiChrom^TM^ calcium ion detection kit according to the manufacturer’s protocol. All the tests were carried out in triplicate, and the average values were shown in the form of tables and graphs.

### 2.4. Cytotoxicity Assay

The cytotoxicity of HAp-PEI and HAp was evaluated by MTT assay. Human pancreatic cancer cell PANC-1, BXPC-3, CFPAC-1 and human pancreatic ductal epithelial cell HPDE6-C7 were seeded in 96-well plates (San Diego, BD American) at a density of 5000 cells/well and incubated for 24 h. HAp-PEI and HAp of various concentrations were added in medium and incubated for another 48 h. Then, 10 μL of MTT (5 mg/mL in pH 7.4 PBS) was added in the medium and incubated for 4-h, after which the medium was replaced with 150 μL DMSO to solubilize the formazan crystals. The UV absorbance intensity of cells, which can be converted to the number of viable cells, was measured by Microplate Reader (TECAN, Männedorf, Switzerland) at λ = 490 nm.

### 2.5. siRNA Loading and Transfection

#### 2.5.1. siRNA Loading

The complexes of HAp-PEI/siRNA were formed by incubating siRNAs with HAp-PEI at room temperature for 20 min after gentle mixing for 10 times. The loading efficiency of siRNA to HAp-PEI was evaluated by agarose gel electrophoresis. The 2 μg of siRNA negative control (siNC) was added into 0, 2, 4, 8, 12, 16, 20, 24 μg of HAp-PEI suspension in DEPC H_2_O, respectively (Table 1). Afterwards, the supernatant was loaded into the wells of 1% agarose gel. The images were acquired using a gene genius bioimaging system.

#### 2.5.2. siRNA-FAM Transfection

PANC-1 cells were seeded on a cell petri dish at a concentration of 2 × 10^4^ cells/well in 2 mL medium and then incubated overnight. Then, siRNA-FAM (green fluorescence), siRNA-Mate/siRNA-FAM and HAp-PEI/siRNA-FAM (the weight ratio of HAp-PEI and siRNA is 8/1) in DEPC H_2_O were added to the cells and incubated for 8 h by keeping 5 μL siRNA-FAM in each group. siRNA-Mate was utilized as a commercial siRNA transfection reagent that served as a positive control compared with the HAp-PEI carrier. Further, the inversed fluorescent microscope (IFM) (Olympus, Japan) was used to confirm intracellular uptake, and lysosomes staining of Lysotracker^TM^ Red (red fluorescence) was employed to locate siRNA-FAM. Briefly, after siRNA transfection for a predetermined time, cells were washed 3 times with PBS followed by the addition of 50 nM Lysotracker^TM^ Red (lysosomes maker). After incubation for 30 min at 37 °C, the cells were washed 3 times with PBS and then observed under a fluorescent microscope, with excitation and emission of green (Ex/Em 494/530 nm for siRNA-FAM) and red (Ex/Em 577/590 nm for Lysotracker^TM^ Red) fluorescence.

#### 2.5.3. KRAS siRNA Transfection

Pancreatic cancer cells (PANC-1 and CFPAC-1) were seeded in 6-well plates at a density of 2 × 10^4^ cells/well and incubated overnight. The cells were treated with HAp-PEI/siKras in the culture medium while other wells were treated with DEPC H_2_O, free siKras, HAp-PEI, HAp/siKras, siRNA-Mate/siKras at equivalent siRNA concentration for 48 h, respectively. The cellular levels of Kras mRNA and protein were assessed using RT-qPCR and Western blot, respectively.

### 2.6. Real-Time Quantitative PCR

Pancreatic cancer cells (PANC-1 and CFPAC-1) were collected, and total RNA from transfected cells was isolated using a total RNA extraction reagent (Genpharm, Shanghai, China). The relative expression of Kras mRNA was detected by a custom gene RT-qPCR quantitation kit (Shanghai, China). The sense primer sequence of *KRAS* gene was 5′-AAC TTG TGG TAG TTG GAG C-3′, and the antisense primer sequence was 5′-GGA TCA TAT TCG TCC ACA AAA TG-3′. Similarly, the sense primer sequence of *GAPDH* was 5′-CAT GAG AAG TAT GAC AAC AGC CT-3′, and the antisense primer sequence was 5′-AGT CCT TCC ACG ATA CCA AAG T-3′. The standard curves were generated, and relative gene expression of *KRAS* was calculated by the 2^−ΔΔCt^ method using *GAPDH* as the reference gene.

### 2.7. Western Blot

The transfected PANC-1 and CFPAC-1 cells were washed twice with PBS and then were lysed by 60 μL of lysis buffer with 1 mmol/L phenylmethanesulfonylfluoride for 30 min on ice. Total protein was acquired after centrifuging at 12,000 rpm at 4 °C for 5 min, and the concentration of protein was determined by BCA protein assay kit (Thermo Scientific, Waltham, MA, USA). The total soluble protein was separated on SDS-PAGE and then transferred to the nitrocellulose membrane. After incubation in primary mouse anti-Kras antibody (Abcam, Cambridge, UK) at 1:1000 dilutions and secondary antibody HRP-labeled Goat Anti-Mouse IgG (H + L) at 1:500 dilutions in succession, the specific blot was visualized using enhanced chemiluminescence substrate (Tanon5500, Shanghai, China).

### 2.8. In Vitro Anti-Pancreatic Tumor Cell Assay

The viability of HAp-PEI/siKras treated pancreatic cancer cells (PANC-1, CFPAC-1 and BXPC-3) was evaluated by MTT assay as described. The cells were seeded in 96-well plates at a density of 5000 cells/well and incubated for 24 h. The cells were treated with HAp-PEI/siKras, DEPC H_2_O, free siKras, HAp-PEI, HAp/siKras, siRNA-Mate/siKras, and HAp-PEI/siNC at equivalent siRNA concentrations, respectively, for 72 h and then their viabilities were evaluated.

### 2.9. Statistical Analysis

Statistical analysis was determined using Student’s paired *t*-test, and the value of *p* < 0.05 was considered statistically significant. All presented data were as the mean ± SD.

## 3. Results and Discussion

### 3.1. Characterization of HAp-PEI

**Figure 1:**
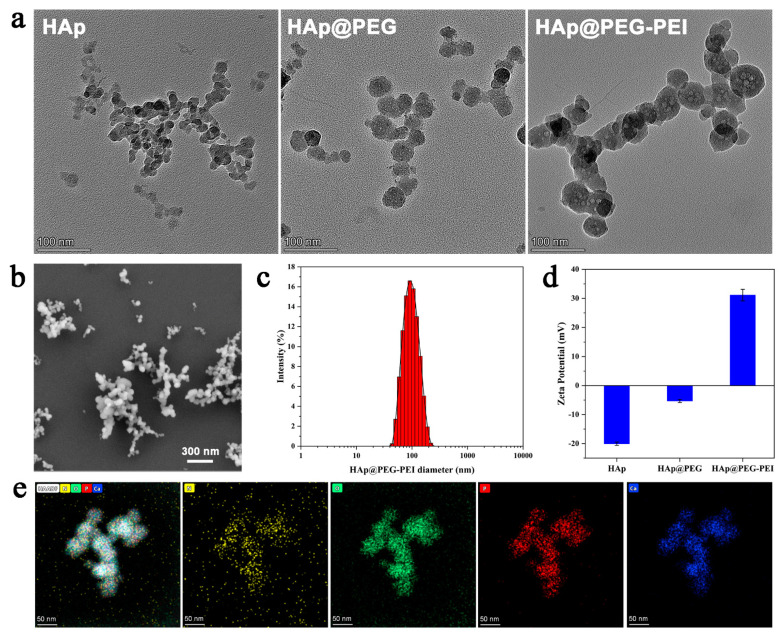
(**a**) TEM images of HAp, HAp@PEG and HAp@PEG-PEI, (**b**) SEM image, (**c**) hydrodynamic size distributions, (**d**) zeta potential, and (**e**) TEM elemental color mapping of HAp@PEG-PEI. Elements: N (yellow), O (green), P (red) and Ca (blue).

HAp-PEI nanoparticles were synthesized with the regulation of PEG and then modified by PEI. Structural characterizations were observed by TEM (Figure 1a) and FE-SEM (Figure 1b), and results showed that pure HAp, HAp with PEG regulation (HAp@PEG) and then PEI-modified NPs (HAp@PEG-PEI) are all in spherical shape. TEM images showed HAp nanoparticles with homogeneous size, and the dispersion of the particles was obviously improved after PEG treatment. HAp@PEG and HAp@PEG-PEI NPs revealed a more uniform and slightly larger size as compared with pure HAp. DLS measurements (Figure 1c) indicated a narrow, monomodal particle distribution with an intensity average hydrodynamic diameter of about 95.76 nm (PDI 0.198) for the HAp@PEG-PEI sample. Moreover, the zeta-potential (Figure 1d) of the prepared PEG-coated HAp NPs changed from −20.03 ± 0.55 mV to −5.30 ± 0.57 mV, and a significant increase was found in the HAp@PEG-PEI sample, indicating that the PEI modification has an obvious effect on the surface of NPs by electrostatic interaction between the negatively charged HAp@PEG NPs and positively charged PEI, which can be beneficial to deliver siRNA with high negative surface charge into the cells for siRNA-based targeted gene silencing. To examine elemental confirmation of the prepared HAp@PEG-PEI NPs, STEM element analysis was achieved and the results are depicted in Figure 1e. The corresponding element mapping analysis clearly endorses the construction of NPs mainly composed of N (yellow), O (green), P (red) and Ca (blue), which further confirms the successful PEI-modification on HAp NPs.

**Figure 2:**
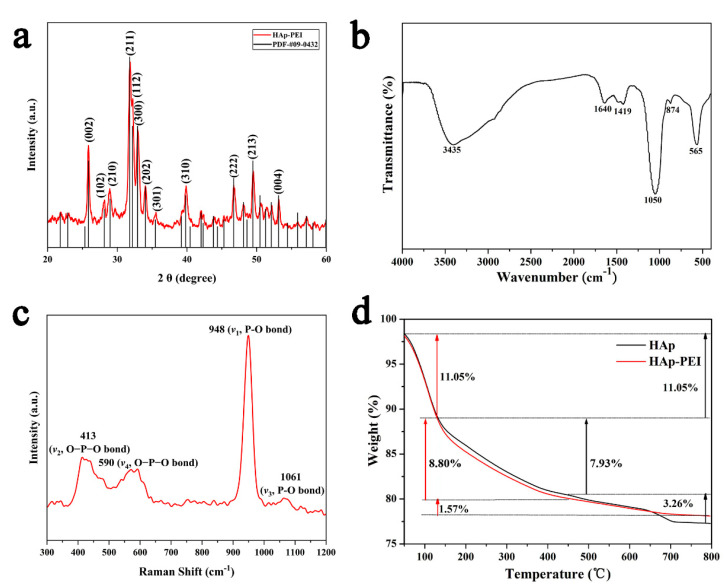
(**a**) XRD pattern, (**b**) FTIR and (**c**) Raman spectra of HAp-PEI, and (**d**) TG curves of HAp and HAp-PEI.

Furthermore, the XRD technique was used to observe the crystal phase and for purity identification of the prepared HAp-PEI NPs. Figure 2a shows the crystal patterns of HAp-PEI. The diffraction spectra of the prepared calcium phosphate are well matched with the XRD standard card of hydroxyapatite (JCPDS#09-0432), and the sharp diffraction peaks indicate the good crystallinity and the hydroxyapatite phase purity. Further, the average crystalline size of HAp-PEI NPs was calculated by the Scherrer equation to be about 36.1 nm. The lattice parameters of HAp-PEI NPs were calculated and the obtained values were a = b = 9.421, c = 6.896 Å, which are consistent with the standard values. In addition, the functional groups in HAp-PEI were characterized by FTIR spectra (Figure 2b). The intense absorption band located at 1050 cm^−1^ is ascribed to the stretching vibration (*ν*_3_) of the phosphate (PO_4_^3−^) groups, and the absorption band located at 565 cm^−1^ is attributed to the bending vibration (*ν*_4_) of the PO_4_^3−^ groups [31,32]. The absorption bands located at 3445 cm^−1^ and 1640 cm^−1^ are attributed to the hydroxyl group and adsorbed water, respectively. Moreover, the appearance of CO_3_^2−^ absorption peaks at 874 cm^−1^ (*ν*_2_) and 1419 cm^−1^ (*ν*_3_) indicates the obtained HAp-PEI is a typical carbonated calcium phosphate [33,34]. Moreover, the HAp-PEI NPs were investigated by Raman spectroscopy. The Raman spectrum (Figure 2c) of HAp-PEI has a characteristic very strong PO_4_^3−^ peak at 948 cm^−1^ (*ν*_1_, P-O bond), which corresponds to a totally symmetric stretching mode of the tetrahedral. Apart from this peak, 413 cm^−1^ (*ν*_2_, O-P-O bond), 590 cm^−1^ (*ν*_4_, O-P-O bond), and 1061 cm^−1^ (*ν*_1_, P-O bond) peaks were also resolved. All bands are assigned to internal vibrational modes of the PO_4_^3−^ groups [35]. The Raman spectrum confirms the XRD pattern and FTIR spectra. The mass loss curve of the HAp-PEI was further carried out by TG analysis, and the curve is roughly divided into three steps (Figure 2d). For HAp and HAp-PEI, the weight loss of 11.05% in the heating process from 20 °C to 130 °C is due to the volatilization of adsorbed water. The weight loss of 7.93% in HAp at 130–450 °C corresponds to the decomposition of the PEG. The weight loss of 8.80% in HAp-PEI is indicating that the polymer content (PEG and PEI) in HAp-PEI is around 8.80%. The difference between HAp and HAp-PEI is about 0.87%, which may be due to the modification of HAp by PEI. The mass loss resulting from further heating represents the decomposition of the hydroxyapatite itself.

### 3.2. In Vitro Degradation of HAp-PEI

**Figure 3:**
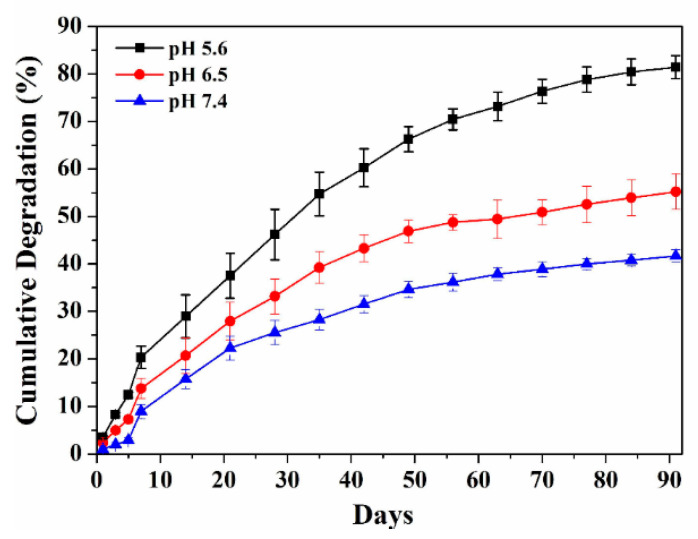
In vitro degradation profiles of HAp-PEI in PBS with various pH values of 5.6, 6.5 and 7.4 at 37 °C.

In vitro degradation of HAp-PEI was performed by immersing in PBS with various pH values of 5.6, 6.5, and 7.4 under gentle shaking at 37 °C for 90 days. The particles need to be degraded quickly after going inside the lysosomes (pH 5.6) to release the siRNA and activate the siRNA-based gene silencing in cancer cells. Therefore, in vitro degradation behavior of HAp-PEI in PBS with pH values of 5.6 was performed. The degradation behavior under pH 6.5 and 7.4 was additionally investigated to confirm the pH-dependent biodegradability of HAp. The degradation behavior for 90 days was to check the long-term behavior of the prepared NPs. As shown in Figure 3, the in vitro degradation behavior of HAp-PEI exhibited a similar trend at different pH values.

After a 90-day degradation profile, the cumulative degradation amount of HAp-PEI in PBS with pH of 5.6, 6.5, and 7.4 reached about 80%, 50%, and 40%, respectively. It is obvious that the degradation rate of HAp-PEI increases significantly with a decrease in pH from 7.4 to 5.6, indicating a sustained and pH-responsive degradation behavior. Therefore, the HAp-PEI nanoparticles possess excellent in vitro degradation ability and could be a promising siRNA carrier for siRNA-targeted gene silencing applications.

### 3.3. In Vitro Biocompatibility of HAp-PEI

**Figure 4:**
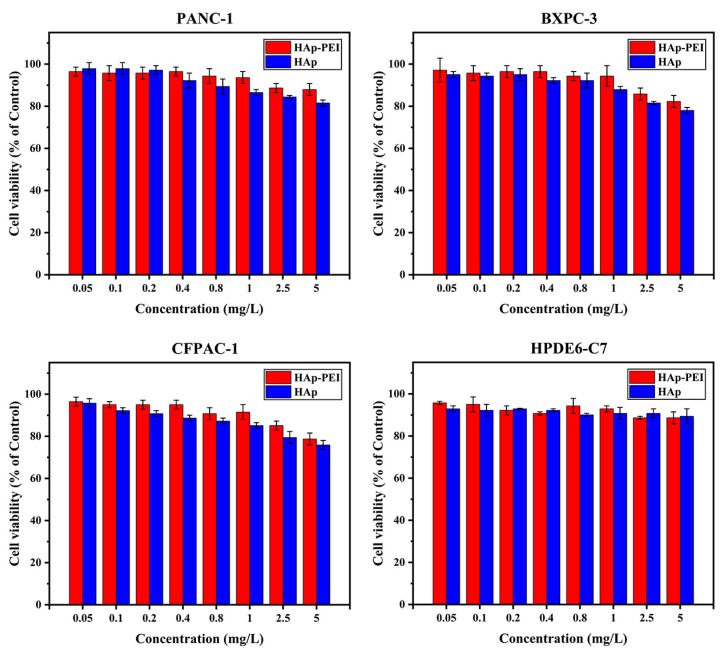
Cell viability of pancreatic cancer cells (PANC-1, BXPC-3, CFPAC-1) and pancreatic ductal epithelial cells (HPDE6-C7) co-incubated with HAp-PEI and HAp at various concentrations (0.05 mg/L–5 mg/L) for 48 h.

Further, in vitro biocompatibility of HAp and HAp-PEI was investigated by MTT assay. The cytotoxicity in human PC cells (PANC-1, BXPC-3, and CFPAC-1) and human pancreatic ductal epithelial cell HPDE6-C7 was evaluated. As shown in Figure 4, MTT assay of cells with various concentrations (0.05 mg/L–5 mg/L) of HAp and HAp-PEI treatment revealed that cell viability compared with control was over 80%, indicating that the HAp and HAp-PEI demonstrate good biocompatibility on both human PC and normal cells, which lays the foundation for its use as an anticancer gene carrier.

### 3.4. siRNA Loading Capability

**Figure 5:**
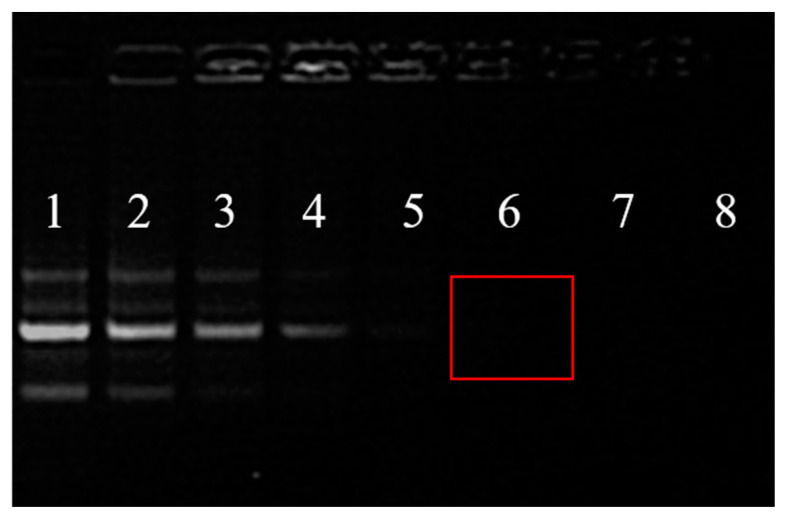
Agarose gel electrophoresis map of siRNA in different groups of siRNA loading. The mass of siRNA was 2 μg, and HAp-PEI were 0, 2, 4, 8, 12, 16, 20, and 24 μg, respectively, in 1 to 8 groups. The weight ratios of HAp-PEI and siRNA (HAp-PEI/siRNA) were 0, 1/1, 2/1, 4/1, 6/1, 8/1, 10/1, and 12/1, respectively, in 1 to 8 groups. Group 1–5: siRNA bands are gradually shallow; Red rectangular marked group 6: HAp-PEI/siRNA (*w/w*) = 8/1, siRNA band has disappeared.

The agarose gel electrophoresis was performed to investigate the siRNA loading capability of HAp-PEI. As shown in Figure 5, with the increment in HAp-PEI concentration, siRNA bands are significantly shallow, and the siRNA band has disappeared when the mass of HAp-PEI is up to 16 μg (group 6, red rectangular marked), which represents that siRNA had been completely adsorbed by HAp-PEI. Thus, the siRNA could be loaded efficiently by HAp-PEI, and the best weight ratio of HAp-PEI and siRNA for loading was 8/1.

### 3.5. In Vitro Transfection of siRNA

**Figure 6:**
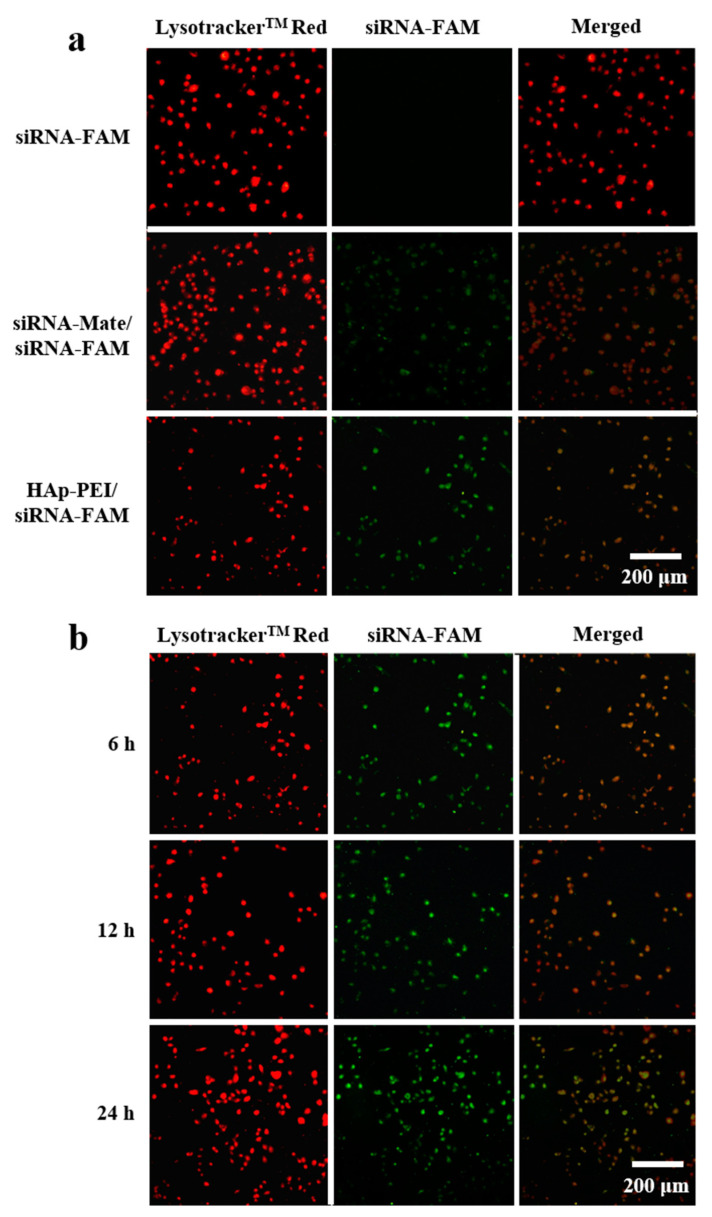
The pancreatic cancer cell PANC-1 transfected siRNA-FAM (green fluorescence) by (**a**) siRNA-Mate and HAp-PEI for 8 h and (**b**) HAp-PEI for 6, 12, and 24 h. Cell lysosome was stained with Lysotracker^TM^ Red (red fluorescence).

In vitro transfection is closely related to siRNA delivery efficiency [36]. In general, high transfection of siRNA leads to the high efficiency of gene silencing. In this study, a fluorescent microscope was used to observe the intracellular uptake of siRNA-FAM (green fluorescence) in PANC-1 cells. As shown in Figure 6a, free siRNA-FAM could not enter into cells due to its negative charge, demonstrating no green fluorescence. Compared with the transfection effect of commercial transfection reagent (siRNA-Mate), siRNA-FAM could be transfected effectively by HAp-PEI and showed no significant difference. Moreover, the green fluorescence (siRNA-FAM) and red fluorescence (lysosome, staining of Lysotracker^TM^ Red) reached almost overlapping, indicating that HAp-PEI has successfully entered into the lysosome of cells after carrying siRNA-FAM, which provides strong support for the RNA interference in cancer cells. Significant visible fluorescence (Figure 6b) is observed after 6-h transfection and existed after 24 h. In addition, the intensity of the fluorescence in HAp-PEI/-siRNA-FAM treatment groups increased as the incubation time prolonged from 6 h to 24 h, suggesting the transfection of siRNA guided by HAp-PEI is effective and lasts for a very long time, which will facilitate siRNA-based gene silencing in cancer cells.

### 3.6. KRAS Gene Silencing Effect

*KRAS* gene mutation has a strong relationship with the incidence of PC, and various strategies have been developed to inhibit *KRAS* gene expression to counteract the growth of pancreatic cancer cells. Earlier researchers reported that many designed nanoparticles served in gene and siRNA delivery. Diverse methods such as covalent chemical bonding have been applied to modified nanoparticles to improve gene and siRNA transfection efficiency. However, the typical chemical approach requires tedious cross-linking procedures with organic chemical solutions, which may destruct the gene and siRNA feature, and affect gene therapy function. We have developed a biocompatible HAp-PEI nanoparticle as an efficient carrier to deliver KRAS-siRNA for anti-pancreatic cancer therapy by a simple and effective electrostatic interaction strategy. In this study, KRAS siRNA (siKras) was transferred into pancreatic cancer cells by HAp-PEI, and the HAp-PEI/siKras based gene-silencing effect was evaluated by RT-qPCR and Western blot assay.

**Figure 7:**
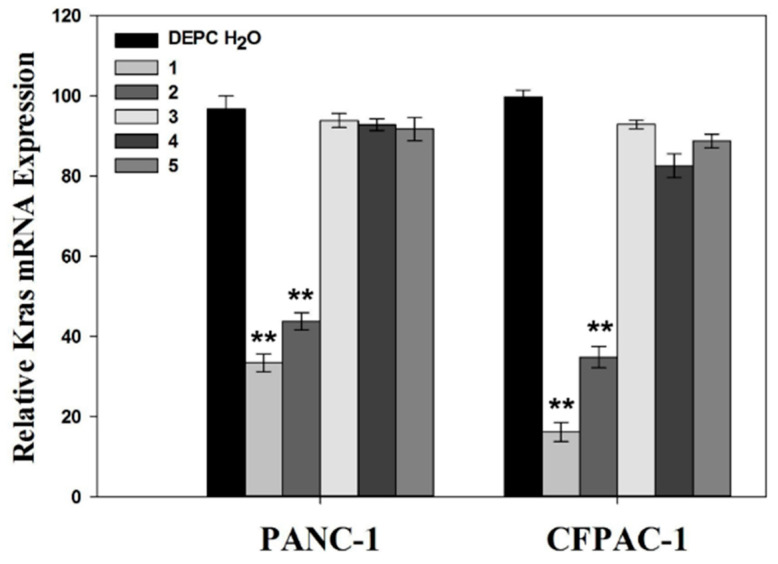
Expression of Kras mRNA in PANC-1 and CFPAC-1 cells after treatment by (1) siRNA-Mate/siKras, (2) HAp-PEI/siKras, (3) siKras, (4) HAp-PEI, and (5) HAp/siKras for 48 h. DECP H_2_O was used as control. Values are represented as mean ± SD (n = 3). Statistical significance relative to the group of control: ** *p* < 0.01.

The prepared HAp-PEI was utilized to deliver siKras into PC cells (PANC-1 and CFPAC-1) and knock down the expression of the *KRAS* gene. After siKras transfection by HAp-PEI and siRNA-Mate for 48 h, the expression of Kras mRNA was evaluated by RT-qPCR. Figure 7 (1. siRNA-Mate/siKras, 2. HAp-PEI/siKras group) revealed that the expression of Kras mRNA is significantly downregulated in PANC-1 cells and CFPAC-1 cells (*p* < 0.01), while *KRAS* gene silencing failed in the DECP H_2_O and (3) siKras, (4) HAp-PEI, and (5) HAp/siKras groups. It is obvious that free siKras (3. siKras group) showed no significant effect of KARS gene silencing (*p* > 0.05), which is consistent with the fluorescence siRNA-FAM transfection result. As predicted, the (2) HAp-PEI/siKras group showed a stronger ability to silence the Kras mRNA expression compared with the (5) HAp/siKras group, indicating that the modification of PEI could greatly increase the siRNA transfection efficiency by pure HAp.

**Figure 8:**
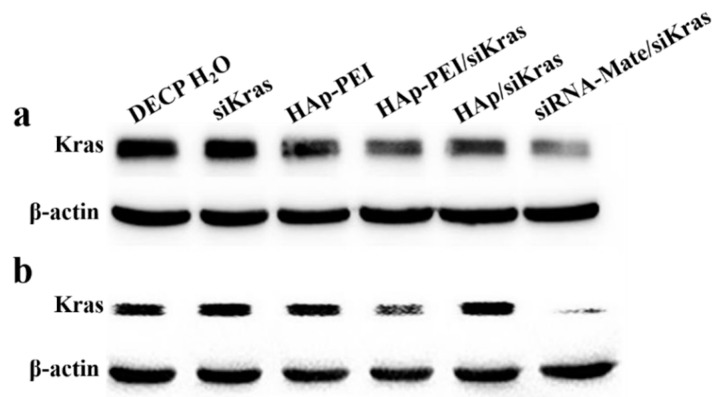
Expression of Kras protein in (**a**) PANC-1 and (**b**) CFPAC-1 cells after siKras transfection for 48 h by HAp-PEI, Hap, and siRNA-Mate. DECP H_2_O, siKras and HAp-PEI treatment were used as control.

Next, the expression level of Kras protein in PANC-1 and CFPAC-1 cells was evaluated via Western blot assay after different treatments to clarify the gene silencing potency of HAp-PEI/siKras. As shown in Figure 8, compared with other groups, the siRNA-mate/siKras group and HAp-PEI/siKras group showed significant downregulation of the expression level of Kras protein. ImageJ software was used to have a relative quantitative analysis of Kras protein expression level. DECP H_2_O treatment was used as a control. As listed in Table 2, the decrease of Kras protein expression level in both PANC-1 and CFPAC-1 cells showed a trend with siRNA-mate/siKras > HAp-PEI/siKras > HAp/siKras, while other treatments, including free siKras, did not display significant influences, which is also consistent with the downregulation expression of Kras mRNA as shown in Figure 7. The results of both RT-qPCR and Western blot demonstrated that the HAp-PEI/siKras could significantly silence the expression of the *KRAS* gene. Therefore, HAp-PEI, as a powerful carrier, is promising for siRNA-based gene silencing in anti-pancreatic cancer.

### 3.7. In Vitro Anti-Pancreatic Cancer Effect

**Figure 9:**
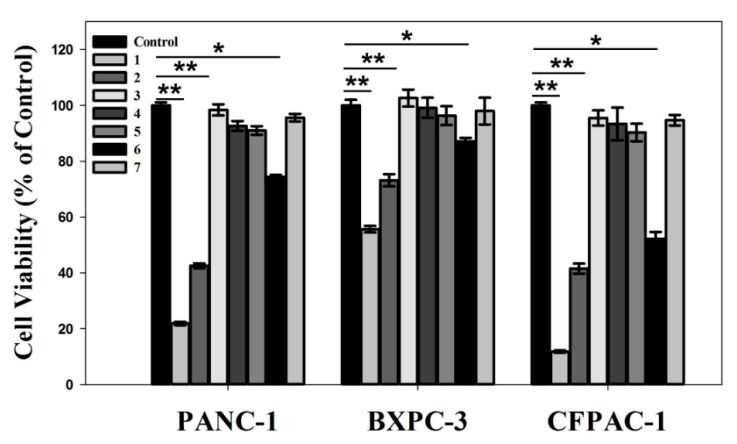
In vitro anti-pancreatic cancer effect. PANC-1, BXPC-3, and CFPAC-1 cell viability after treatment by (1) siRNA-Mate/siKras, (2) HAp-PEI/siKras, (3) DEPC H_2_O, (4) siKras, (5) HAp-PEI, (6) HAp/siKras, and (7) HAp-PEI/siNC for 72 h. Untreated cells were used as control. Values are represented as mean ± SD (n = 3). Statistical significance relative to the group of control: * *p* < 0.05, ** *p* < 0.01.

*KRAS* gene silencing through RNA interference can inhibit the growth of pancreatic cancer cells, which is promising for gene therapy of pancreatic adenocarcinoma disease. The prepared HAp-PEI/siKras in this study is proven to have an effective *KRAS* gene silencing effect (Figure 7 and Figure 8). Thus, we evaluated the in vitro anti-pancreatic cancer effect in PC cells (PANC-1, BXPC-3 and CFPAC-1) via MTT assay. Cells were treated with (1) siRNA-Mate/siKras, (2) HAp-PEI/siKras, (3) DEPC H_2_O, (4) siKras, (5) HAp-PEI, (6) HAp/siKras, and (7) siNC/HAp-PEI, respectively, for 72 h. Untreated cells served as the control groups. According to the MTT assay results, the cell viabilities in HAp-PEI/siKras-treated PANC-1, BXPC-3and CFPAC-1 cells were about 40%, 65%, and 40%, respectively, in comparison with the untreated cells, suggesting significantly high cytotoxicity of PC cells (Figure 9, *p* < 0.01). The HAp-PEI/siKras nanoparticles degraded quickly after going inside the lysosomes (pH 5.6) to release the siRNA and activated the siRNA-based gene silencing in cancer cells, which shows positive results in mRNA and protein expression level, and pancreatic cancer cells were killed. These results may confirm the stability of siRNA in different physiological conditions. Moreover, the cytotoxicity of HAp and HAp-PEI in the normal pancreatic cell HPDE6-C7 evaluated by MTT is shown in Figure 4, and the cell viability remained over 90%, indicating their low toxicity and side effects.

## 4. Conclusions

In this work, we developed a biocompatible HAp-PEI nanoparticle as an efficient carrier to deliver *KRAS*-siRNA for anti-pancreatic cancer therapy. The active tumor-targeted HAp-PEI/siKras nanoparticle exhibited high siRNA transfection efficiency comparable to a commercial transfection reagent siRNA-Mate, effectively knocked down the expression of *KRAS* gene, and downregulated the expression of Kras protein in vitro. Meanwhile, HAp-PEI/siKras was able to inhibit the PC cells’ (PANC-1, BXPC-3 and CFPAC-1) proliferation. Moreover, no obvious in vitro cytotoxicity was observed of HAp-PEI-treated normal pancreatic cell HPDE6-C7, suggesting its good compatibility in such a gene delivery system. Finally, these studies provided a potential strategy of siRNA-targeted gene therapy for anti-pancreatic cancer therapy.

## Figures and Tables

**Table 1:** Amounts of HAp-PEI and siRNA with the different weight ratios.

Groups	1	2	3	4	5	6	7	8
HAp-PEI/siRNA (*w*/*w*)	/	1/1	2/1	4/1	6/1	8/1	10/1	12/1
HAp-PEI (μg)	0	2	4	8	12	16	20	24
siRNA (μg)	2	2	2	2	2	2	2	2

**Table 2:** Relative quantification of Kras protein expression.

	DEPC H_2_O	siKras	HAp-PEI	HAp-PEI/siKras	HAp/siKras	siRNA-Mate/siKras
PANC-1	100%	97.65%	96.46%	51.52%	74.13%	22.22%
CFPAC-1	100%	106.15%	104.03%	67.04%	105.10%	32.87%

## Data Availability

Not applicable.
